# Alternative technologies in cervical cancer screening: a randomised evaluation trial

**DOI:** 10.1186/1471-2458-6-252

**Published:** 2006-10-16

**Authors:** Ahti Anttila, Matti Hakama, Laura Kotaniemi-Talonen, Pekka Nieminen

**Affiliations:** 1 Mass Screening Registry, Finnish Cancer Registry, Liisankatu 21 B, FI-00170 Helsinki, Finland; 2 School of Public Health, FI-33014 University of Tampere, Tampere, Finland; 3 Department of Obstetrics and Gynaecology, Helsinki University Central Hospital, Box 140, FI-00029, Helsinki, Finland

## Abstract

**Background:**

Cervical cancer screening programmes have markedly reduced the incidence and mortality rates of the disease. A substantial amount of deaths from the disease could be prevented further by organised screening programmes or improving currently running programmes.

**Methods/Design:**

We present here a randomised evaluation trial design integrated to the Finnish cervical cancer screening programme, in order to evaluate renewal of the programme using emerging technological alternatives. The main aim of the evaluation is to assess screening effectiveness, using subsequent cancers as the outcome and screen-detected pre-cancers as surrogates. For the time being, approximately 863,000 women have been allocated to automation-assisted cytology, human papillomavirus (HPV) DNA testing, or to conventional cytology within the organised screening programme. Follow-up results on subsequent cervical cancers will become available during 2007–2015.

**Discussion:**

Large-scale randomised trials are useful to clarify effectiveness and cost-effectiveness issues of the most important technological alternatives in the screening programmes for cervical cancer.

## Background

Conventional pap smear screening has reduced effectively incidence and mortality rates of cervical cancer in several countries [1-5]. The rates vary still greatly between countries. Along with the background risk, variation in the rates depends largely upon availability and also quality of screening.

During the last decade new screening methods have been developed. However, validation of the new technological options has almost entirely relied on intermediate parameters, such as screen-detected findings; in many cases only on the numbers in cytological findings. Those evaluations interfere with serious limitations, due to large variation in the rates of screen-detected lesions and also their regression potentials. Therefore invasive cervical cancers diagnosed after screening or prevented by screening has been proposed as the standard for assessing screening validity [6]. Along with sensitivity, specificity; or, e.g. test or treatment rates; should be compared with numbers of prevented cancers. If the numbers of tests or treatments increase, but there prove to be no clearly better impact, then the new technique is not better than the conventional one. Considering the long duration of pre-cancerous lesions – averaging 10–12 years [6] – this possibility can be clarified only with rather long-lasting follow-up studies.

We introduce here a randomised screening design to evaluate technological alternatives in cervical cancer screening. The design and corresponding protocol have been developed and integrated in the Finnish cervical cancer screening programme. The design enables early large-scale implementation, and is aiming by that means to evaluate the ultimate effectiveness of the screening programmes with the technological alternatives. We also report the current status of this evaluation trial, discuss the most updated results available thus far on the intermediate markers, and discuss conditions relevant for comparative studies in other European countries. The latter aspect is important when considering possibilities for joint analyses.

## Methods and design

### Effectiveness conventional screening

In Finland (population 5.2 million; target population 1.2 million, women in ages 30–64 years) a nation-wide effective programme for cervical cancer screening has been in action for four decades. Currently the age-adjusted death rate from cervical cancer is about 1/100,000 woman-years (national average during 1999–2003, annual world-standardised rate) – more than 85% lower than the level before screening [7]. Among the five similarly developed Nordic countries (overall population 25 million) with variable screening practices [8] about 1000 deaths are avoided per year; however, nearly 1500 could be avoided with optimal screening [9].

**Figure 1:**
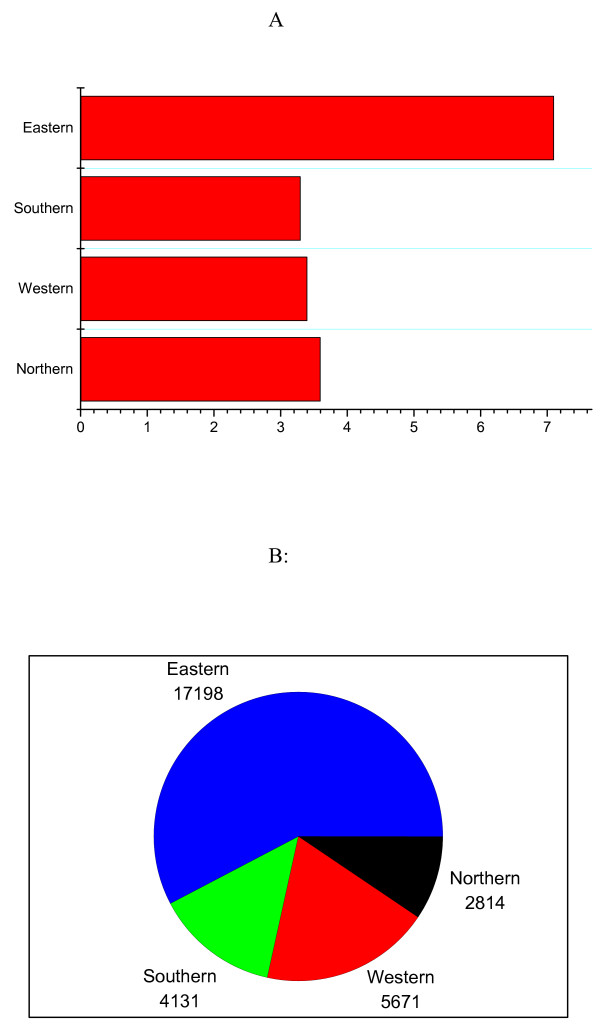
Estimated age-standardised death rates and numbers of deaths from cervical cancers in European regions (Globocan 2002); a) Age-adjusted (ASR(W)) mortality rate from cervical cancer in Europe, and b) Numbers of deaths from cervical cancer in Europe.

In the whole Europe, there are five-fold differences in the current cervical cancer rates between countries [10,11]. About 60,000 incident cervical cancer cases are diagnosed annually, and some 30,000 deaths are estimated to take place from the disease (Figure 1). Some countries lack cervical cancer screening totally whereas an organised national programme is running only in a minority of the countries [6,8]. There are no systematic studies available on screening effectiveness in whole Europe, due to lack of historical information on screening and in many areas on cervical cancer.

### Alternative screening techniques and information available on their sensitivity

Over the years several new technologies have been proposed for screening: automation-assisted cytology [12-14], liquid-based cytology (LBC) [15,16], and HPV-DNA testing [17-21]. More recently, HPV-mRNA [22,23] and tests based on the integration of HPV in the genome or cell regulation (such as p16^INK4A^) [6,24] have been proposed as a screening tests, or for triaging or confirmation of progressive lesions. Vaccination against HPV infections and cervical neoplasia will become a new option for prevention and will likely affect screening programmes [25-27].

For a single Pap smear screening test, a false negative result for any cervical intraepithelial neoplasia (CIN) or worse varies between 30–70% [28,29]. A proportion of these lesions regress spontaneously [6,30]. The sensitivity of the Pap test is quite good to detect lesions that would progress invasive within few years from testing, sensitivity among screened is about 80–90% [31].

In an early randomised study, automation-assisted screening has been reported to have almost a twofold detection rate of invasive cervical cancer and CIN3 in comparison with conventional cytology [12]. However, this was not confirmed in a later study [32], where the detection rate was similar between the two methods. HPV-DNA test method has been suggested possibly to be more sensitive than the Pap smear, but with lower specificity [6]. On LBC, there are some 60 cross-sectional studies suggesting similar or higher detection rates [16].

### Purpose of the evaluation trial

The cross-sectional validity information available on the alternative screening techniques interfere with serious limitations, due, e.g., to regression potentials largely unrecognised. The purpose of the current randomised evaluation trial is to evaluate efficacy and effectiveness of the alternative screening techniques using cancer incidence and mortality follow-up as the gold standard. Along with sensitivity, also specificity and corresponding test and treatment rates will be compared between the screening methods. For the current Finnish study, randomised evaluations have been chosen to take place for automation-assisted cytological screening and for primary screening with an HPV-DNA test.

### Ethical and medico-legal aspects

Conventional cytological screening for cervical cancer, including histological confirmation and colposcopy-directed treatments, has been proven effective and it is considered applicable as a public health policy [6,33]. The structure of the screening programme with invitations, screening tests, histologically confirmed pre-cancerous lesions and their management enables introduction of test modifications or other similar components under safe conditions. Safety is maintained by cross-sectional or longitudinal pilot studies preceding a large-scale randomised evaluation phase, and by monitoring the cross-sectional screening findings during the run of the programme. Systematic monitoring and quality assurance will be done according to specifically defined protocols for each technology [6,33]. In a randomised study, this includes defining stopping rules.

The infrastructures and settings for organised screening as well as for randomised screening within the organised programme vary a lot between countries. In Finland, individual randomisation has been conducted while drawing the invitations from population files. There is a continuous linkage between invitation, screening and cancer registry files, based on the national legal framework for organised screening and for data collection within health care. Informing women takes places under the normal practice within the screening programme. For HPV screening, further efforts have been done to train sample-taking nurses within the activity area, including how to communicate with women; as well as a brief leaflet produced to be attached with the invitation letter to screening. The woman has a right to refuse from the HPV-DNA test; in such as case the conventional smear will be performed. Written informed consent for registration purpose is not required. The protocols for the current randomised screening have been approved by the Ethical Committee of the National Research and Development Centre for Welfare and Health (STAKES, 4151/54/98), by the Ethical Committee of the Obstetrics and Gynaecology in Hospital District of Helsinki and Uusimaa (221/E8/02), by the National Authority for Medicolegal Affairs (3950/32/300/02), and by other health-care authorities [14,21,36]. Informing women, as well as data registration has been organised in accordance with directive on personal data registration and corresponding national legislation (95/46/EC)[34,35].

### Protocol in brief

In Finland, women in ages 30 to 60 are invited to the cervical cancer screening programme, in some municipalities in addition women of ages 25 and/or 65; and in case of a normal result the invitational interval is 5 years. Referral to colposcopy and biopsy takes place after clearly positive cytology (Papanicolaou groups III-V; equivalent to LSIL+ according to the Bethesda 2001 classification) or after repeated borderline findings (group II, equivalent to ASC-US) based on the recommendation by the cytologist. Women will be assigned either to the intervention arm or to the conventional screening arm within the area of the participating cytology laboratories when processing the invitational data to the programme. In automation-assisted screening, this information will be made available through the screening notification cards passed to the cytology laboratories together with the screening slides. When indicated, the laboratories scan and analyse the slides using the automation-assisted method (Papnet), and also record along with the intention also the method which was actually used [14,32,36]. Otherwise the screening process is similar than in the conventional practice.

In the HPV screening arm, the sample-taking nurse collects the conventional cytological sample, and the HPV-DNA test sample will be produced thereafter from the endocervical subsample by placing the tip of the sampler brush of the kit to the transport medium. Primarily, the HPV-DNA test sample will be analysed in the cytology laboratory using high-risk HPV Hydrid Capture 2 assay (Digene Corporation). If the HPV-DNA test is positive (the relative light unit ratio ≥ 1.00), then the pap smear will be analysed as a triage for investigating whether the woman needs directly a referral [20,21]. If the HPV-DNA test is positive but the cytology negative, the woman will be re-invited after a year.

### Study size estimates

Available estimates on sensitivity error make basis for calculating of statistical power and study size. In Finland, the absolute rate of invasive cervical cancer after screening cytologically negative within the programme is 5.2 per 100,000 woman-years [37]. There is no good data on current CIN3+ incidence available, but we estimate latter to be about four-fold to the invasive cancer rate.

The subsequent cancer rate is affected by background risk, age groups targeted, quality and history of screening, and follow-up data quality. In the Nordic countries, variation in the cervical cancer incidence rates in time before screening was up to two-fold in comparison with the Finnish ones [38] and one cannot rule out even more variation in a larger geographical area [10,39]. Somewhat higher disease rate estimates (10, 15, 20 cases per 100,000) than reported for the subsequent invasive cervical cancer incidence from the Finnish programme were therefore also used.

In study size estimates we used a test for differences in screening effects [40]. We assumed that if the proposed improvements in screening sensitivity were true, about 50% improvement in screening efficacy could be maximally obtained, in comparison with conventional screening, and that 30% or no improvement of sensitivity might be realistic alternatives as well. Respectively, equivalence – where the required study size is likely to be larger [41] – could also be considered depending upon the method. Calculations presented are valid for any intervention, including HPV vaccination, among women screened negative.

The minimum population size, required for a marginal effect of 50% in further reducing subsequent cervical cancer incidence, varies from 0.3 to 1.0 million woman-years (followed among those attending screening; alpha 0.05; power 80%; two-sided). This assumes that attendance rate is unaffected. There may be drop-outs also due to technical difficulties, or unwillingness of the invited women to have the allocated test; which can be considered consequently. Verifying a marginal decrease of 30% necessitates, respectively, about 2 to 3 fold materials (Table 1).

## Results

The feasibility phase started in Finland in 1997 and the first randomised screening activity in 1999 by introducing the automation-assisted technique. In 2003 primary HPV-DNA testing for high risk HPV types (with Hybrid Capture 2) was introduced. During 1999–2005 approximately 860,000 women have been randomised (table 2). In areas with automation-assisted screening the sampling ratio has been 1:2 (and 1:1:1 in areas with HPV screening included, from 2003 onwards). First reports on screening detection rates are available [14,21,32,36]. Screening detection rates in automation-assisted screening are very similar to conventional screening. Based on very early results (about 5,000 women randomised during 2003) the detection rate of mild pre-cancerous lesions was in excess in the HPV screening protocol. The first report on the interval cancer incidence in automation-assisted screening is expected during the course of 2006–2007, and final analyses of incidence of invasive cancer and CIN3+ later on 2010–2015, including HPV screening.

After two screening rounds (5–9 follow-up years by birth-cohort) the statistical power of the Finnish study will be sufficient to find a rather small (50%) marginal effect in the invasive cancer incidence (automation-assisted screening) or CIN3+ incidence (HPV screening) between the compared technologies.

## Discussion

Public health policies are evaluated often at an ecological level. Randomised settings offer possibilities for optimal evaluation, however [42]. There are few other examples of cancer screening programmes with randomisation. In Finland the nationwide breast cancer screening was implemented in 1980s by a randomised design [43], and randomised implementation of colorectal cancer screening started in 2004 [44].

Conventional cytological screening, including confirmation and treatment, is considered applicable as a public health policy based upon evidence available up to invasive cervical cancer endpoints [6,33]. Similar information is not available for the new methods. The European Union recommends a programme to use conventional Pap smears, even though modifications are acceptable. The recommendation does not guide in detail how de novo programmes or new techniques should be implemented and evaluated. The IARC work group recommends, on the other hand, that only rather restricted demonstration projects need to precede large-scale implementation of new test methods, including HPV-DNA test, in cervical cancer screening programmes; and that the large-scale implementation needs to be designed so as to allow long-term evaluation with randomised designs.

Decisions when or if to implement a new technique have differed greatly. In many countries, some LBC methods are already used routinely whereas many other countries are awaiting for results from RCT-type randomised trials [45,46]. As to HPV-DNA testing, there are several options how to integrate the test [45,46]. In primary screening one need to decide whether to use HPV-DNA test as a sole test, or as an additional test to cytology.

Unlike cytological test, HPV-DNA test does not indicate who is in the immediate need of confirmation and treatment. After a positive HPV-DNA test, cytological information is still required to find the right person for colposcopical examination [47]. Sole HPV-DNA test might lead to many unnecessary colposcopies, because most of the infections will regress rapidly without causing significant cellular atypia. Currently, the treatment decisions are based on the histological confirmation obtained from colposcopy-directed biopsies and the treatments are done with aid of the colposcope. Thus pap smears can be used as a triage after a positive HPV-DNA test to define the need of colposcopy [20,21]. Under the above conditions also HPV-DNA testing can be considered as a modification to the screening programme and thus it is suitable for a randomised evaluation trial phase.

In countries presently without any screening, organising an effective programme is an urgent need. These countries, many of them in the Eastern Europe, need to consider which technology to use in organising their cervical cancer screening. Laboratory, training & education, and other such demands differ a lot between test technologies. Screening as adopted in the well-to-do health care systems is not optimal as to their cost-effectiveness and certainly not for a different or low-resource environment [48]. Targeted screening age groups and intervals, and lifetime numbers of tests and treatments differ between screening policy and by technology options, contributing total cost-effectiveness. Particularly, no good observational information exists on how many tests lifetime are required in a primary HPV screening programme.

Usual time frame required from the start of feasibility phase up to the final information on the efficacy and effectiveness of new technology is about twenty to thirty years. With efficient collaboration and uniform designs this time can be shortened up to about ten years. The randomised implementation of screening results in more rapid evidence of new methods and spontaneous implementation will do.

To conclude, running a randomised evaluation trial within a screening programme, where the experimental arm – the exposure – is the public health policy with new technology and the control arm is the old policy, is a feasible approach for screening programmes for cervical cancer. Current information on validity of various competing screening methods does not directly relate to occurrence of interval cancers. Potential for improved sensitivity and effectiveness, but instantaneously also the cost-effectiveness aspects and potential harm need to be taken into consideration when considering the public health policies organised with the technological alternatives.

## Competing interests

The author(s) declare that they have no competing interests.

## Authors' contributions

AA is currently the primary investigator of the study, and has been responsible author of the paper. MH was formerly the primary investigator (retired), and has originally proposed the concept of the randomised screening evaluation. LKT has participated in the coordination and statistical analysis and writing of this paper. PN serves as the chief medical consultant of the study, and has contributed to the study performance and coordination, as well as writing. All authors read and approved the final manuscript.

## Pre-publication history

The pre-publication history for this paper can be accessed here:



## Figures and Tables

**Table 1:** Study size to obtain 80% statistical power in the proposed screening interventions for different baseline rates for invasive cervical cancer incidence assuming various marginal effects, when α = 0.05 and intervention to control ratio 1:1

Invasive cervical cancer incidence per 10^5 ^woman-years^1^	RR	Woman-years in follow-up (intervention group); 1-sided test	Woman-years in follow-up (intervention group); 2-sided test
5	0.5	820,000	1,020,000
5	0.7	2,467,000	3,097,000
10	0.5	410,000	510,000
10	0.7	1,233,000	1,548,000
15	0.5	273,000	340,000
15	0.7	822,000	1,032,000
20	0.5	205,000	255,000
20	0.7	617,000	774,000

Calculation assumes inclusion of all the ages within organised screening for cervical cancer.

^1 ^In Finland the baseline rate for invasive cervical cancer incidence is approximately 5, in the combined European material from 10 to 20

**Table 2:** Current and estimated study size in the randomised screening design for cervical cancer, Finnish programme during 1999–2008.

	1999–2005		Overall 1999–2008	
	Invited women	Attended (estimate)	Invited (estimate)	Attended (estimate)

Randomised	862,800	609,100	1,000,000	706,000
Automation-assisted	262,900	185,300	301,900	212,800
HPV-DNA	50,800	35,600	99,600	70,000
Conventional	549,100	388,200	598,500	423,200
